# Deposition of Nanostructured CdS Thin Films by Thermal Evaporation Method: Effect of Substrate Temperature

**DOI:** 10.3390/ma10070773

**Published:** 2017-07-09

**Authors:** Nafiseh Memarian, Seyeed Mohammad Rozati, Isabella Concina, Alberto Vomiero

**Affiliations:** 1 Faculty of Physics, Semnan University, Semnan, 35131-19111, Iran; 2 Department of Physics, University of Guilan, Rasht 41335, Iran; smrozati@gmail.com; 3 Division of Materials Science, Department of Engineering Sciences and Mathematics, Luleå University of Technology, 97187, Luleå, Sweden; isabella.concina@ltu.se (I.C.); alberto.vomiero@ltu.se (A.V.)

**Keywords:** CdS thin films, transparent conducting layers, sputtered thin films, optical and electrical properties of thin films

## Abstract

Nanocrystalline CdS thin films were grown on glass substrates by a thermal evaporation method in a vacuum of about 2 × 10^−5^ Torr at substrate temperatures ranging between 25 °C and 250 °C. The physical properties of the layers were analyzed by transmittance spectra, XRD, SEM, and four-point probe measurements, and exhibited strong dependence on substrate temperature. The XRD patterns of the films indicated the presence of single-phase hexagonal CdS with (002) orientation. The structural parameters of CdS thin films (namely crystallite size, number of grains per unit area, dislocation density and the strain of the deposited films) were also calculated. The resistivity of the as-deposited films were found to vary in the range 3.11–2.2 × 10^4^ Ω·cm, depending on the substrate temperature. The low resistivity with reasonable transmittance suggest that this is a reliable way to fine-tune the functional properties of CdS films according to the specific application.

## 1. Introduction

Cadmium sulfide (CdS) is a compound semiconductor comprising group II–VI elements. CdS has a relatively wide band gap (E_g_ = 2.42 eV. at room temperature), and is an intrinsic n-type semiconductor [1]. These properties make it a very desirable window layer for many heterojunction thin film solar cells, such as those based on Cu_2_S [2], CdTe [3], CuInSe_2_ [4]. This promising material is also applied in a wide variety of other fields, such as light emitting diodes [5], photonic devices [6,7,8], photoconductive sensors [9] and environmental pollution control [10]. 

Different methods have been reported for the deposition of CdS thin films, including vacuum evaporation [11,12], chemical bath deposition (CBD) [13,14], closed space sublimation (CCS) [15], spray pyrolysis [16,17], successive ionic layer adsorption and reaction (SILAR) [18], pulsed laser deposition (PLD) [19,20,21] and sol-gel [22]. Each technique produces films with different properties, which should be optimized for specific applications. Since non-vacuum techniques for thin film deposition are inherently more susceptible to oxidation and contamination, vacuum deposition techniques are more suitable for CdS film preparation [9]. These methods are convenient for preparing pinhole free, homogenous and smooth thin films with the required thicknesses. Pulsed laser deposition (PLD) and thermal vacuum evaporation (also known as vacuum evaporation) are two vacuum-based methods for preparation of CdS thin films. 

The influence of temperature on the structural and functional properties of CdS thin films has been investigated in the recent past [23,24,25]. However, in previous studies on this topic, only a limited number of temperatures were selected, impairing the prediction of the best treatment temperature for suitably tuning the optical and electrical properties of thin films.

In this work, we applied vacuum evaporation for the preparation of CdS thin films. We investigated the effect of substrate temperature during film growth on the structural and functional properties of the samples in the temperature range 25–250 °C, with a maximum temperature interval of 50 °C between two consecutive samples. This way, we demonstrated the possibility of fine-tuning the optical and electrical properties of the films in a rather broad range of absorbance and resistivity, precisely identifying the correspondence between deposition temperature and functional properties.

## 2. Results and Discussion

### 2.1. Morphological Properties

**Figure 1:**
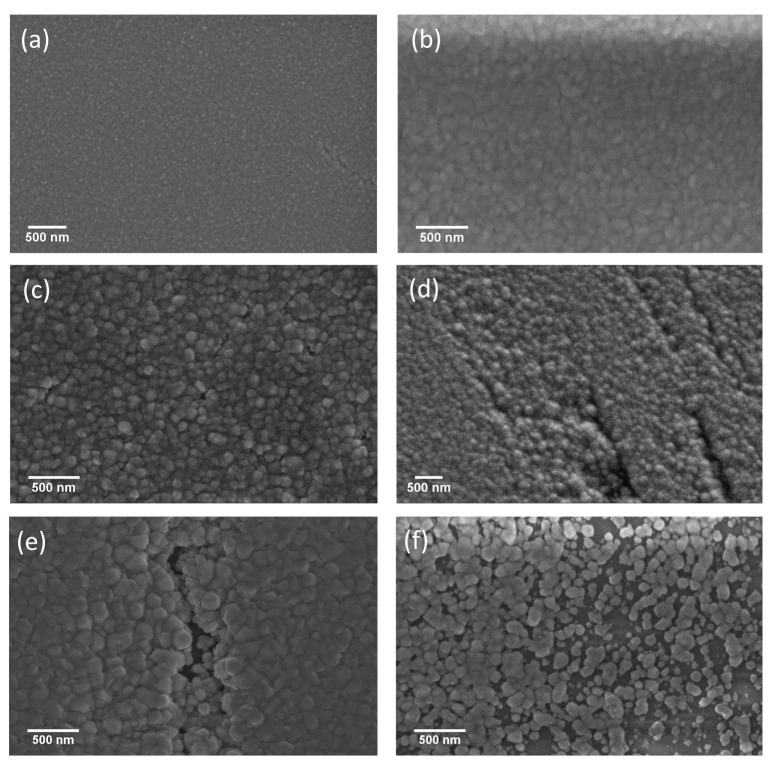
SEM images of nanostructured CdS thin films deposited at different substrate temperatures: (**a**) 25 °C; (**b**) 50 °C; (**c**) 100 °C; (**d**) 150 °C; (**e**) 200 °C and (**f**) 250 °C.

Figure 1 shows SEM images of nanostructured CdS thin films deposited at different substrate temperatures. From SEM images, it is observed that the CdS films deposited up to 150 °C are uniform, without cracks and have a dense surface morphology that covers the entire substrate surface area. Small nano-sized grains with homogeneous and well-defined grain boundaries are uniformly distributed. At first, by increasing the deposition temperature from room temperature (Figure 1a) to 50 °C (Figure 1b) the grain size increases, whereas by further increasing the substrate temperature to 150 °C (Figure 1d), some non-uniformity and small cracks are observed on the sample surface. Additional increase of the deposition temperature to 200 °C (Figure 1e) results in the cracks becoming deeper. Finally, for the film deposited at 250 °C, a non-homogeneous coverage of the substrate has been obtained, with formation of agglomerated clusters.

### 2.2. Structural Properties

**Figure 2:**
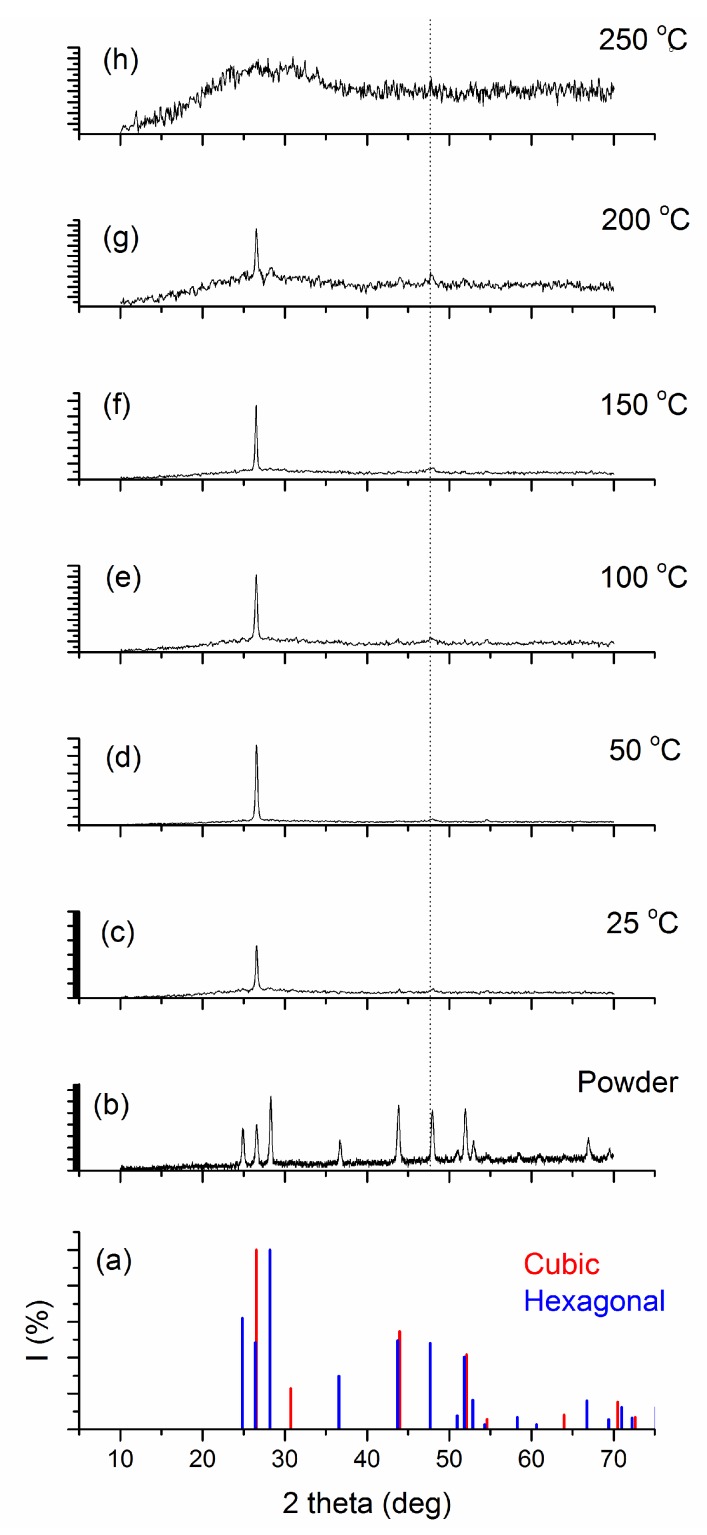
XRD patterns of the (**a**) standard JCPDS cards for hexagonal and cubic structure; (**b**) source CdS powder and vacuum-evaporated CdS thin films deposited at different substrate temperatures: (**c**) 25 °C; (**d**) 50 °C; (**e**) 100 °C; (**f**) 150 °C; (**g**) 200 °C and (**h**) 250 °C.

The X-ray diffraction (XRD) technique allows accurate determination of the crystal structure of materials. It has been reported that the preferred orientation of thin film on glass substrate is affected by experimental parameters in different deposition technique [26,27]. Diffractograms of the CdS source powder and the films, prepared at different substrate temperatures, are shown in Figure 2. 

XRD analysis for the source CdS powder showed that the powder has a polycrystalline hexagonal structure (JCPDS card 96-900-8863) with different orientations. No trace of cubic CdS is detected in the powder (JCPDS card 96-900-8840). The intensity of peaks in powder was smaller than deposited films. XRD analysis showed that the deposited films have highly oriented crystallites with hexagonal structures (Wurtzite type, which is the most thermodynamically stable [23]) and preferential orientation along the c-axis ((002) direction) perpendicular to the substrate plane, irrespective of substrate temperature. A very weak peak at 2θ = 47.92° (dashed line in Figure 2), corresponding to the (013) plane [28], is also observed in the XRD pattern of deposited films, which confirms the hexagonal phase of the thin film, ruling out the presence of cubic phase. Elemental cadmium and/or CdO are not present in the diffractograms, suggesting that oxidation is prevented during thin film growth. Furthermore, raising the substrate temperature did not lead to the formation of other phases. 

The calculated structural parameters, including interplanar distance (d), crystallite size (D), dislocation density (δ) and microstrain (ε), are presented in Table 1 (for the calculations, see detailed description in the Materials and Methods section). The interplanar distance is calculated using Bragg’s law. 

For the film deposited at 250 °C the XRD shows completely amorphous structure so no structural parameter is included in the Table 1 for that sample. The small values of δ obtained in the present study confirm the good crystallinity of the thin films fabricated by the thermal evaporation technique. 

It is observed that the crystallinity of the films initially increased with substrate temperature, reaches the maximum grain size of 26 nm at 50 °C and thereafter it goes on decreasing with increase in substrate temperature. The initial increase in crystallinity and crystallite size with substrate temperature is due to the optimum rate of supply of thermal energy for recrystallization with substrate temperature [29]. In fact, the film prepared at 50 °C has a better crystalline quality, as indicated by the corresponding XRD pattern. With further increase in substrate temperature, there is a decline in peak intensity. For the film deposited at 250 °C, a completely amorphous structure is eventually observed. The best structural properties (i.e., lowest dislocation density and highest crystalline size) are found in the film deposited at 50 °C. It is interesting that the crystallographic orientation of the films did not change by increasing the substrate temperature. In Ulrich’s work [30] for CdS deposition by the PLD method with different laser emissions, tuning of the orientation of crystalline domains was only observed with the 355 nm PLD, and not with 1064 nm. Thermally driven deposition and 1064 nm PLD induced the same behavior during thin film growth as for the orientation of crystalline domains, while UV PLD resulted in different processes of film nucleation and growth. Apparently a cluster-free (i.e., atom per atom deposition) deposition, irrespective of the deposition method, results in (002) oriented CdS films. The form and energy of the impinging particles in combination with the thermal energy provided by the substrate defines the end product; this also explains amorphous structure of the film deposited at 250 °C.

### 2.3. Optical Properties

**Figure 3:**
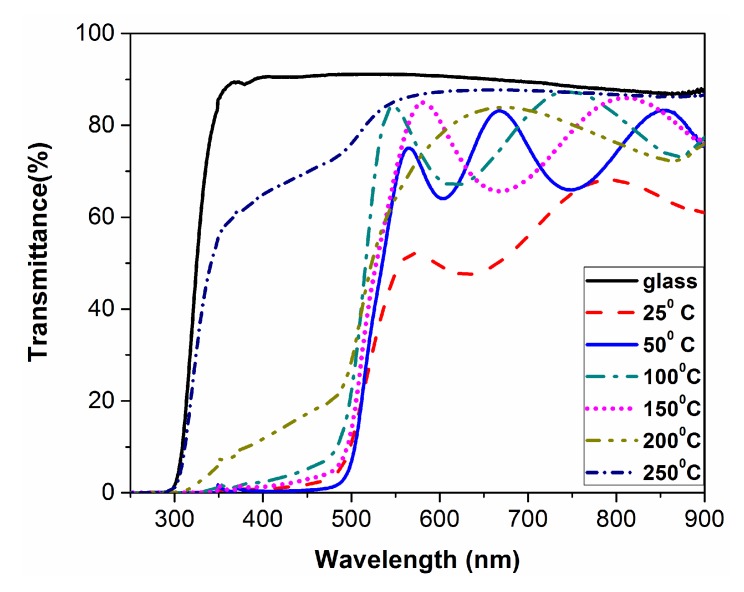
Optical transmittance spectra of CdS thin films deposited at different substrate temperatures.

Figure 3 shows the transmittance of CdS films deposited at different substrate temperatures. The oscillations of transmittance in the visible region are due to thin film interference effects. By increasing the substrate temperature from 25 °C to 50 °C, there is a sharp rise in transmittance, which remains almost constant up to 200 °C. For higher substrate temperatures (200 °C and 250 °C) the spectra exhibit non-negligible UV features, which reflect inhomogeneous substrate coverage (also highlighted in SEM micrographs) and the amorphous behavior of the films. 

The evaporated CdS particles in the high temperature sample (250 °C) have more kinetic energy, so they may spread around and are not deposited uniformly on the substrate, according to SEM and XRD.

The film thicknesses are listed in Table 2. According to Table 2, the film formed at 25 °C had a thickness of 450 nm, i.e., 110 nm less than the film formed at 50 °C. Nevertheless, the average transmittance of the film formed at 25 °C is about 15% less than the one formed at 50 °C. Although at first glance this is a contradiction, the reason is obvious: the film formed at 25 °C possesses the highest reflection because of the smooth surface (as is clear in the SEM image Figure 1a).

### 2.4. Electrical Properties

Sheet resistance, resistivity and thickness of the films are listed in Table 2. The sharp increase in the resistivity of vacuum-deposited CdS thin films by increasing the deposition temperature from 50 to 100 °C is in good agreement with the results of other research groups [31]. 

This strong dependence of resistivity on substrate temperature could be due to the alterations of the stoichiometry of the films. It has been shown by SIMS [28] that evaporated films possess a Cd surplus, which is responsible for the n-type conductivity. This Cd surplus of the films deposited at 25 °C and 50 °C could be the cause of a relatively high conductivity, considerably lowering the resistivity. 

Although the film deposited at 25 °C has the lowest resistivity, it is not the best film in general for practical purposes, because its average transmittance in the range 550–800 nm is about 55%, lower than the other films, making it unsuitable for some functional applications such as the window layer of solar cells. Therefore, the film deposited at 50 °C with relatively low resistivity (3.3 Ω·cm) and acceptable average transmittance (about 78%) is the best film in this set of experiments. 

**Figure 4:**
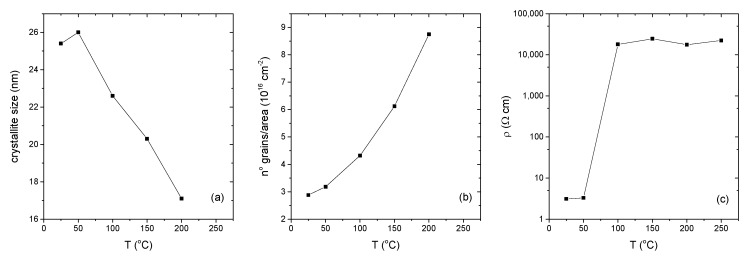
Structural and electrical properties of the CdS thin films as a function of substrate temperature. (**a**) Grain size; (**b**) number of grains per unit area; (**c**) electrical resistivity. Solid lines are a guide for the eye.

In Figure 4, the main structural and functional features are collected, giving an overview at a glance of the main parameters of the produced thin films as a function of the substrate temperature.

## 3. Materials and Methods

CdS powder with 99.995% purity from Sigma Aldrich (St. Louis, MO, USA) was used as the source material. Thin films of CdS were deposited at different substrate temperatures, ranging from room temperature to 250 °C in steps of 50 °C, on-to pre-cleaned glass substrates, at a base pressure of 2 × 10^−5^ Torr using a Hind Hi-Vac 15F6 Model vacuum coating unit (Bangalore, India). The evaporation was carried out by resistive heating of the CdS powder from a molybdenum boat. Substrate to source distance was 15 cm. All the parameters were kept constant during the 10 min deposition. 

The surface morphology of the films was investigated using scanning electron microscopy (SEM), carried out with a LEO 1525 microscope (Westchester County, NY, USA). The thickness of films was measured by using Alpha-step IQ KLA (Milpitas, CA, USA) Tencor surface profilometer. 

Philips Analytical XRD PW-1830 (Amsterdam, The Netherlands) diffractometer using CuK_α_ radiation was used for X-ray diffraction measurements. 

Crystallite size was estimated by using Scherrer’s formula given by Equation (1) [32]

(1) $$D=\frac{0.9\;\lambda }{\beta \;\cos\theta }$$

where, D is the crystallite size, λ is the wavelength of the radiation used (1.54 Å) and β is full width at half of the peak maximum (FWHM). The dislocation density (δ), defined as the length of dislocation lines per unit volume, was estimated using the following Equation [33,34]:

(2) $$\delta =\frac{1}{D^{2}}$$

δ is the measure of the number of defects in a crystal. The number of crystallites per unit area (N) and the strain (ε) of the films were determined with the use of the following Formulae [35]:

(3) $$N=\frac{t}{D^{3}}$$

(4) $$\varepsilon =\frac{\beta \;\cos\theta }{4}$$

where (t) is the thickness of the film.

Electrical resistivity was measured by a four-point probe method using a Keithley 2410 current source (Cleveland, OH, USA), electrometer and nano-voltmeter using Van der Pauw configuration. To evaluate the optical properties of films, UV–VIS spectroscopy (Cary 100 Scan Varian, Ames, IA, USA) were carried out.

## 4. Conclusions

The semiconducting thin films of CdS were successfully deposited on glass substrates using the vacuum evaporation technique at various substrate temperatures ranging from 25 °C to 250 °C. The CdS films exhibit a hexagonal structure with strong orientation along (002) direction, irrespective of substrate temperature, and no mixed phases were observed. It is observed that the crystallinity initially increased with substrate temperature, but thereafter continued to decrease. The grain size was between 17 and 26 nm. Scanning electron microscopy studies revealed uniform deposition up to 150 °C. For higher substrate temperatures (200 °C and 250 °C), the transmittance spectra pattern and, therefore, the film thickness was changed, resulting in increased transparency in the short wavelength region, most probably because of the formation of cracks. The electrical resistivity of CdS films strongly increased as substrate temperature increased above 50 °C. These results indicate that CdS films of low resistivity and high transmittance can be produced by vacuum evaporation at low substrate temperatures, and can be profitably used for several applications, including solar cells, light emitting diodes and photonic devices in general.

## Figures and Tables

**Table 1:** Structural parameters of CdS thin films.

Substrate Temperature (°C)	Crystallite Size, D (nm)	d (Å)	No. of Grains/Area N (×10^12^) (cm^−2^)	Dislocation Density δ (lines/m^2^)	Strain, ε (×10^−^^3^)
25	25.4	3.347	2.75	1.60 × 10^15^	1.36
50	26.0	3.357	3.18	1.48 × 10^15^	1.35
100	22.6	3.357	4.32	2.06 × 10^15^	1.53
150	20.3	3.355	6.12	2.50 × 10^15^	1.71
200	17.1	3.357	8.75	3.46 × 10^15^	2.02

**Table 2:** Electrical parameters of CdS thin films. t: film thickness; R_sh_: sheet resistance; ρ: resistivity.

Substrate Temperature (°C)	t (nm)	R_sh_ (Ω/□)	ρ (Ω·cm)
25	450	69 × 10^3^	3.11
50	560	59 × 10^3^	3.30
100	460	390 × 10^6^	1.79 × 10^4^
150	490	500 × 10^6^	2.45 × 10^4^
200	430	410 × 10^6^	1.76 × 10^4^
250	360	623 × 10^6^	2.24 × 10^4^
